# OsWRKY26 negatively regulates bacterial blight resistance by suppressing *OsXa39* expression

**DOI:** 10.3389/fpls.2024.1519039

**Published:** 2025-01-09

**Authors:** Win Tun, Kieu Thi Xuan Vo, Behnam Derakhshani, Jinmi Yoon, Lae-Hyeon Cho, Kay Tha Ye Soe Win, Sang-Won Lee, Ki-Hong Jung, Jong-Seong Jeon, Gynheung An

**Affiliations:** ^1^ Graduate School of Green-Bio Science, Kyung Hee University, Yongin, Republic of Korea; ^2^ Department of Biological Sciences and Bioengineering, Industry-Academia Interactive R&E Center for Bioprocess Innovation, Inha University, Incheon, Republic of Korea; ^3^ Department of Plant Bioscience, College of Natural Resources and Life Science, Pusan National University, Miryang, Republic of Korea

**Keywords:** disease, OsWRKY26, *OsXa39*, rice, *Xanthomonas oryzae*

## Abstract

Plants are susceptible to infection by various pathogens with high epidemic potential. *Xanthomonas oryzae* pv*. oryzae* (*Xoo*) causes bacterial blight in rice, one of the most significant diseases in both temperate and tropical regions. In this study, we report the identification and characterization of *OsWRKY26*, a sucrose-inducible transcription factor, that plays a role in the plant defense responses following *Xoo* infection. We found that mutant plants with defective *OsWRKY26* showed enhanced defense response specifically to *Xoo*, indicating that this transcription factor acts as a negative defense regulator. In contrast, mutant plants did not exhibit higher resistance compared to wild-type (WT) plants when infected with the rice blast fungal pathogen *Magnaporthe oryzae*. Transcriptomic analysis of mutant and WT plants revealed that several pathogen resistance genes were upregulated in mutants. Of these, we selected *OsXa39* for further analysis. Transient expression experiments in rice protoplasts showed that OsWRKY26 repressed the expression of a *Luciferase* reporter gene driven by the *OsXa39* promoter. Chromatin immunoprecipitation analysis revealed that OsWRKY26 binds directly to the promoter region of *OsXa39*. These findings suggest that OsWRKY26 negatively regulates the defense response during *Xoo* infection by repressing *OsXa39* as well as other pathogen-related genes such as *OsXa47*, *OsBBR1*, *OsRSR1*, *OsPR1a*, *OsPR1-11*, *OsPR2*, and *OsPR4c.*

## 1 Introduction

Rice is one of the most important staple food crops throughout the world, but its production is significantly threatened by various pathogens, including bacterial blight *Xanthomonas oryzae* pv*. oryzae* (*Xoo*) (Mondal et al., 2021). In general, plants defend themselves against pathogens via two primary immune mechanisms: pathogen-associated molecular pattern (PAMP)-triggered immunity (PTI) and effector-triggered immunity (ETI) (Vo et al., 2023). PTI acts as an early line of defense by recognizing common pathogen molecules through pattern-recognition receptors (PRRs) located on the membrane, which include receptor-like protein (RLPs) and receptor-like kinases (RLKs). RLKs have a single transmembrane domain to recognize pathogen molecules and intracellular kinase domain for signal transduction. Upon recognition, PRRs activate downstream pathways, including the MAPK cascade, to trigger immune responses such as ROS generation, cell death, and increased cytoplasmic calcium ion concentration (Yu et al., 2024). In contrast, ETI provides a stronger and more specific response by detecting pathogen effectors through intracellular resistance (R) proteins (Yuan et al., 2021; Vo et al., 2023). ETI plays a critical role in *Xoo* resistance, as specific R genes recognize *Xoo* effectors and trigger corresponding defense responses (Zhao et al., 2009; Lee et al., 2011; Fiyaz et al., 2022). Among the R genes, the nucleotide-binding and leucine-rich repeat (NLR) genes, along with variable amino-terminal domains, are particularly important as the family consists of the largest group in rice. These proteins mediate the recognition of transcription-activator-like effectors (TALEs) produced by *Xanthomonas*. TALE effectors facilitate pathogenicity because they induce the expression of susceptibility (S) genes, which are involved in normal plant physiological processes. Moreover, specific manipulations have been found to alter R gene-mediated resistance to *Xanthomonas* (Hammond-Kosack and Kanyuka, 2007; Lee et al., 2011; Nowack et al., 2022; Xu et al., 2022).

The accumulation of R gene transcripts can also confer resistance to specific pathogen strains, while mutations in R genes lead to susceptibility. For example, the R gene *OsXa39*, a member of the NLR gene family, is known to confer resistance to 21 *Xoo* strains. Previous studied using the rice line H471, which carries *OsXa39*, has revealed that it triggers a robust hypersensitive response involving programmed cell death upon infection with *Xoo* strains (Shi et al., 2022; Zhang et al., 2015a, 2015b; Zhang et al., 2015c). These findings highlight its effectiveness in providing durable resistance to a varierty of *Xoo* strains. Knockout mutants of another NLR gene, *Xa47*, are more susceptible to *Xoo*, whereas the overexpression of *Xa47* in the susceptible rice variety JG30 has been found to increase *Xoo* resistance (Lu et al., 2022). Similarly, the overexpression of the NLR gene *OsBBR1*, was demonstrated to confer moderate resistance to the *Xoo* strains PXO86 and PXO341 via upregulation of pathogenesis-related (PR) gene expression (Wang et al., 2013). Comparable resistance phenomena have also been found for the NLR gene *Xa1* and several of its alleles (e.g., *Xa1-2* and *Xa14*), as well as for *Xa31* (Zhang et al., 2020). Although many NLR genes may be involved in resistance to *Xoo*, at present the ways in which these genes are controlled by upstream regulatory elements remain poorly understood.

WRKY transcription factors (TFs) are known to mediate several signaling pathways and regulatory networks involved in defense responses (Xie et al., 2021; Javed and Gao, 2023). For example, in rice a RNAi-mediated knockdown of *OsWRKY6* was found to reduce resistance to *Xoo* infection, and was accompanied by decreased expression of the defense-related genes *OsPR1a* and *OsPR1b* (Im et al., 2022). In addition, the OsWRKY6 protein activates other *WRKY* genes such as *OsWRKY45* and *OsWRKY47* (Im et al., 2022). Recent work has also shown that CRISPR/Cas9 mutations in *OsWRKY7* result in longer lesions in plants infected with *Magnaporthe oryzae* and *Xoo* than is found in wild-type (WT) plants. In contrast, overexpression of this gene is associated with increased expression of several pathogenesis-related (PR) genes, including *OsPR1a*, *OsPR1b*, and *OsPR10a*, which leads to pathogen resistance (Tun et al., 2023; Zheng et al., 2023). In another study, OsWRKY10 was found to enhance resistance to *M. oryzae* via direct activation of diterpenoid biosynthesis genes (Wang et al., 2023). Overexpression of *OsWRKY11* confers resistance to *Xoo* strains by directly binding to the promoter region of *CHITINASE2*, while silencing of the *WRKY* gene results in reduced resistance (Lee et al., 2018). Other studies focusing on ectopic expression of *OsWRKY22* found that it enhanced resistance to *M. oryzae*, whereas knockout mutant lines showed higher pathogen susceptibility (Abbruscato et al., 2012). Furthermore, constitutive activation of *OsWRKY30* enhances defense responses to *Xoo* via mediation of the salicylic acid (SA) signaling pathway (Han et al., 2013). Overexpression of *OsWRKY31* resulted in enhanced resistance to *M. oryzae*, accompanied by increased expression of defense-related genes *PBZ1* and *OsSci2* (Zhang et al., 2008). Moreover, RNA silencing of *OsWRKY67* was found to reduce the levels of SA as well as the expression levels of SA- and PR-associated genes, including *OsPR1a* and *OsPR10a*. This resulted in higher susceptibility to both *M. oryzae* and *Xoo*, whereas overexpressing the *WRKY* gene enhanced resistance to both pathogens (Liu et al., 2018).

Some *WRKY* genes function as negative regulators of plant defense responses to bacterial and fungal pathogens. For example, one study showed that constitutive expression of *OsWRKY28* impaired resistance to *M. oryzae* (Chujo et al., 2013), while another showed that overexpression of *OsWRKY72* led to reduced levels of endogenous jasmonic acid (JA) as well as lowered expression of JA- and PR-related genes, thereby resulting in increased susceptibility to *Xoo* infection (Hou et al., 2019). Similarly, overexpression of *OsWRKY76* has been found to increase susceptibility to *M. oryzae* by reducing the expression levels of PRs and phytoalexin biosynthesis genes via the suppression of W-box elements (Yokotani et al., 2013).

Interestingly, some WRKY TFs seem to have multiple functions. During *Xoo* invasion, *OsWRKY53* functions as a negative regulator by suppressing *OsMYB63*, which strengthens the sclerenchyma cell walls of vascular tissues via the activation of cellulose synthases, including *OsCesA4, OsCesA7*, and *OsCesA9* (Xie et al., 2021). In contrast, OsWRKY53 functions as a transcriptional activator by inducing the expression of PR genes, thus improving resistance to *M. oryzae* (Chujo et al., 2007, 2014). Moreover, overexpression of *OsWRKY62* is known to suppress the expression of several defense-related genes, which results in enhanced susceptibility to infection by both *Xoo* and *M. oryzae* (Peng et al., 2008; Xu et al., 2022). However, when OsWRKY62 forms a heterodimer with OsWRKY45, the dimer functions as a positive regulatory element in inducing diterpenoid phytoalexin biosynthesis genes to enhance defense against pathogens (Fukushima et al., 2016).

In general, WRKY proteins interact with numerous other proteins, such as kinases, receptors, and other transcription factors, to form transcriptional regulatory networks (Chen et al., 2019). These proteins can either stimulate or inhibit the expression of various downstream genes through TGAC core sequences, including the W-box motif, W-box like elements 1 (WLE1), and ASF1MOTIF (Maleck et al., 2001; Hwang et al., 2008). However, some WRKY proteins can also bind to other core sequences within the promoter regions of downstream genes (Van Verk et al., 2008; Machens et al., 2014).

*OsWRKY26*, also referred to as *OsWRKY59* and *DLN19* (https://rapdb.dna.affrc.go.jp; Singh et al., 2019) belongs to the group II WRKY family and is upregulated by *M. oryzae* infection (Li et al., 2021). Interestingly, both compatible and incompatible strains of *Xoo* induce the expression of *OsWRKY26* (Choi et al., 2017), which suggests that it plays a role in pathogen defense. However, to date the molecular mechanisms responsible for its involvement in plant defense responses remain unclear. In this study, we show that *OsWRKY26* functions as a negative regulator of defense against *Xoo* by directly binding to the promoter of *OsXa39*, a known broad-spectrum NLR gene, and suppressing its expression.

## 2 Materials and methods

### 2.1 Plant materials and growth conditions

For all experiments, we used *Oryza sativa* var. *japonica*, cultivar “Dongjin” to generate transgenic lines and act as a WT control. Seeds were germinated on the ½ Murashige and Skoog (MS) medium, supplemented with 3% sucrose and 0.3% agar, and seedlings were maintained in the medium until 10 days after germination (DAG). The plants were then transferred to growth rooms with controlled conditions (14-h-light at 28°C/10-h-dark at 22°C, with relative humidity maintained at 50% –70%), greenhouses, or the paddy field, depending on the experimental requirements.

### 2.2 Vector construction and rice transformation

We generated *oswrky26* mutants by clustered regularly interspaced short palindromic repeats (CRISPR)/CRISPR-associated nuclease 9 (Cas9)-mediated gene editing. To do so, we designed two single guide RNA (sgRNA) targets using two web-based tools: http://crispr.dbcls.jp and http://crispor.org (Naito et al., 2015; Concordet and Haeussler, 2018). The structures of the RNA scaffolds of the target sequences were verified using the bioinformatic tool (http://rna.tbi.univie.ac.at/cgi-bin/RNAWebSuite/RNAfold.cgi). The specificity of target sequences was confirmed using CRISPR RGEN Tools (http://www.rgenome.net/cas-offinder/).

For transformation, synthesized and annealed sgRNA sequences were first ligated into the *Bsa*I-digested pRGEB32 vector (Xie et al., 2015). The resulting plasmids were then transformed into *Agrobacterium tumefaciens* strain LBA4404 using the freeze-thaw method (An, 1987). These were subsequently transformed into rice embryonic calli according to a previously published method (Lee et al., 1999). Transgenic T0 plants were then selected on ½ MS agar medium including 50 mg L^−1^ hygromycin. After selection, genomic DNA was extracted from transformed plants, and the target sequence was PCR amplified using primers flanking the target site. Finally, the resulting PCR products were subcloned into T-easy vectors, and five colonies from each line were sequenced to validate sequence identities.

For the overexpression of *OsWRKY26* in transgenic plants, the full-length cDNA of *OsWRKY26* was amplified by PCR using primers (**Supplementary Table 1**) with leaf cDNA as a template and inserted into the binary vector pGA3438 (Kim et al., 2009) using the 5x In-fusion HD enzyme (STO345, Takara). The resulting construct was then introduced into *A. tumefaciens* LBA4404 and transformed into rice. As a control, transgenic plants expressing the Myc tag alone were also generated. To confirm the expression of the target gene, total protein was extracted from the leaf blades of six transgenic T1 lines at 50 DAG, and exogenous OsWRKY26 was detected by western blotting using a Myc antibody (9B11 with HRP conjugate; Cell Signaling Technology). Additionally, *OsWRKY26* transcript levels were analyzed by qRT-PCR, with *OsUbi5* as the reference gene.

The *OsWRKY26* promoter:*β-glucuronidase* (*GUS*) vector (*pWRKY26:GUS*) was created by insertion of a fragment 3,000 bp upstream of the start codon into a pGA3519 vector (Yoon et al., 2014) through the *Kpn*I and *Hpa*I restriction enzymes sites. *pWRKY26:GUS* transgenic plants were then stained using GUS solution (50 mM sodium phosphate pH 7.0, 1 mM potassium ferricyanide, 1 mM potassium ferrocyanide, 0.1% triton X-100, 10 mM EDTA, pH 8.0, 0.1% X-Glu dissolved in 1% DMSO and 5% methanol). Plants showing a strong GUS signal were selected for further analysis.

### 2.3 RNA isolation and transcript level measurement

RNA was isolated, and transcript levels were quantified following the method as previously reported (Tun et al., 2023). Transcript levels of specific genes were examined at different time points; these levels were normalized to those of rice *Ubiquitin 5* (*OsUbi5*, *LOC_Os01g22490*) (Jain et al., 2006), which was used as an internal control. At least three biological replicates were performed for each time point. Relative expression levels were calculated using the ΔΔCt method; primer specificity was verified by observing a single sharp peak in a melting curve analysis (Schmittgen and Livak, 2008; Cho et al., 2016). The primers used in this study are listed in **Supplementary Table 1**.

### 2.4 Pathogen infection and spore inoculation

*M. oryzae* inoculation was conducted as a previously described method (Tun et al., 2023). Briefly, *M. oryzae* isolate RO1-1 was first grown on V8 juice agar plates (80 mL L^−l^ V8 Juice [Campbell’s Soup Company, Camden, NJ], 15 g L^−1^ agar, pH 6.8) for 2 weeks under continuous fluorescent light. Conidia were collected, examined, and quantified under a microscope, then suspended in water to reach a final concentration of 5 x 10^6^ mL^−1^. Leaves from 50 DAG plants were used for spot inoculation (Kanzaki et al., 2002). Particularly, 3 μl of conidia suspension was applied to 2 mm press-injured spots on the leaves. After inoculation, the infected plants were placed in a closed box to maintain 100% relative humidity at 25°C in the dark for 24 h before being transferred to a moist incubator. Infected leaves were harvested at 9 days post inoculation (DPI) for lesion length quantification and photographed.

The *Xoo* strain PXO99 was used for bacterial infection analyses. *Xoo* inoculation on the leaves were carried out using a previously described leaf clipping method (Ke et al., 2017). For the calculation of colony-forming units (CFUs), leaf fragments (6 cm in length) from below the *Xoo* lesion area were collected at 12 DPI. CFUs were calculated according to the previous method (Ke et al., 2017). Lesion length was measured at 14 DPI to assess the degree of disease symptoms.

### 2.5 Transcriptomic analyses

The second leaves from WT and *OsWRKY26* mutant plants at 50 DAG were used for transcriptome analyses. Total RNA was extracted in triplicates from each line and subjected to RNA sequencing. Sequencing data quality was assessed using FastQC v0.11.9 (http://www.bioinformatics.babraham.ac.uk/projects/fastqc/). To obtain high-quality clean reads, low-quality bases from both 5′ and 3′ ends of the paired-end reads were trimmed using Trimmomatic v0.39 (Bolger et al., 2014). The rice reference genome was downloaded from the Rice Genome Annotation Project database (http://rice.plantbiology.msu.edu/pub/data/Eukaryotic_Projects/o_sativa/annotation_dbs/pseudomolecules/version_7.0/all.dir/) (Kawahara et al., 2013). Index construction for the reference genome and alignment of the cleaned reads to the reference genome were both performed using Hisat2 v2.2.1 using the default parameters (Kim et al., 2015). Aligned SAM files were then converted to BAM format and sorted using SAMtools v1.10 (Danecek et al., 2021). Raw read counts were generated from the alignment files with featureCounts v2.0.0, which is part of the Subread package v1.6.2 (Liao et al., 2014). Post-normalizations of read counts were calculated as Fragments Per Kilobase of transcript per Million mapped reads (FPKM) to facilitate the estimation of gene expression levels of each sample. Differentially expressed genes were then identified using the DEseq2 package v1.38.3 (Love et al., 2014), using an adjusted *p*-value threshold of 0.05 and an absolute log_2_ fold change ≥1. Functional annotations of genes were obtained by performing a BLASTP search against the NCBI Nr protein database with an E-value cutoff of 1.0e-8. Using the MSU7 functional annotation files, description, Gene Ontology (GO) terms, and Pfam IDs were assigned to each gene. GO enrichment analysis of all upregulated genes in the biological process category was carried out using the BiNGO tool, employing a hypergeometric test and Benjamini & Hochberg False Discovery Rate correction to obtain an adjusted *p*-value cutoff of < 0.05 (Maere et al., 2005).

### 2.6 Subcellular localization of OsWRKY26

The cDNA sequence of the *OsWRKY26* coding region was inserted into the *BamH*I and *Kpn*I-digested pGA3452 vector containing the *ZmUbi1* promoter, a *green fluorescence protein* (*GFP*) coding region, and the *nos* terminator (Kim et al., 2009). The resulting fusion construct was then co-transformed with a vector expressing a nuclear localization signal (NLS)-red fluorescence protein (RFP) conjugate under the control of the *ZmUbi1* promoter (NLS-RFP) into protoplasts isolated from rice Oc cells (Baba et al., 1986). Fluorescence signals were observed using a confocal microscope (K1-Fluo, Nanoscope, Korea) 14 h after transformation. GFP and RFP were excited at 488 nm and 561 nm, respectively, and their emission signals were collected using 525–550 nm and 561 nm long-pass filters, respectively.

### 2.7 Transient assay to assess transcriptional activity

The full-length coding sequence of *OsWRKY26* was inserted between the *ZmUbi1* promoter and the HA tag through the *Hpa*I and *Kpn*I restriction enzymes into pGA3698 (Kim et al., 2009) using In-fusion HD enzymes. The resulting vector was used as an effector construct. For luciferase reporter assays, different lengths of the *OsXa39* promoter sequence were subcloned into a pGL4.23 vector that consisted of a minimal promoter and the *Luciferase* coding sequence (E8411, Promega). To normalize luciferase activity, the *ZmUbi : GUS* vector (Cho et al., 2008) was used as an internal control.

### 2.8 Chromatin immunoprecipitation

Chromatin immunoprecipitation (ChIP) experiments were performed as a previously described protocol (Haring et al., 2007). Briefly, fresh leaves from *OsWRKY26-OX-*9 and Myc tag alone transgenic plants were harvested at 10 DAG. Proteins and chromatin complexes were cross-linked with 1% formaldehyde in solution A (10 mM Tris pH 8.0, 400 mM sucrose, 0.1 mM PMSF, and 5 mM β-mercaptoethanol). After 10 min of vacuum infiltration, 125 mM glycine was added to quench the reaction. Following nuclei isolation, extracted DNA was sonicated to fragments of approximately 200-1,000 bp in length. Immunoprecipitation was performed using an anti-Myc antibody (9B11, cell signaling) and agarose beads conjugated with protein G and A (EMD Millipore). Washing and reverse cross-linking steps were then carried out according to the protocol of Haring et al. (2007). The enriched chromatin was analyzed by PCR using the primer sets listed in **Supplementary Table 1**.

## 3 Results

### 3.1 *OsWRKY26* is preferentially expressed in vascular bundles

**Figure 1:**
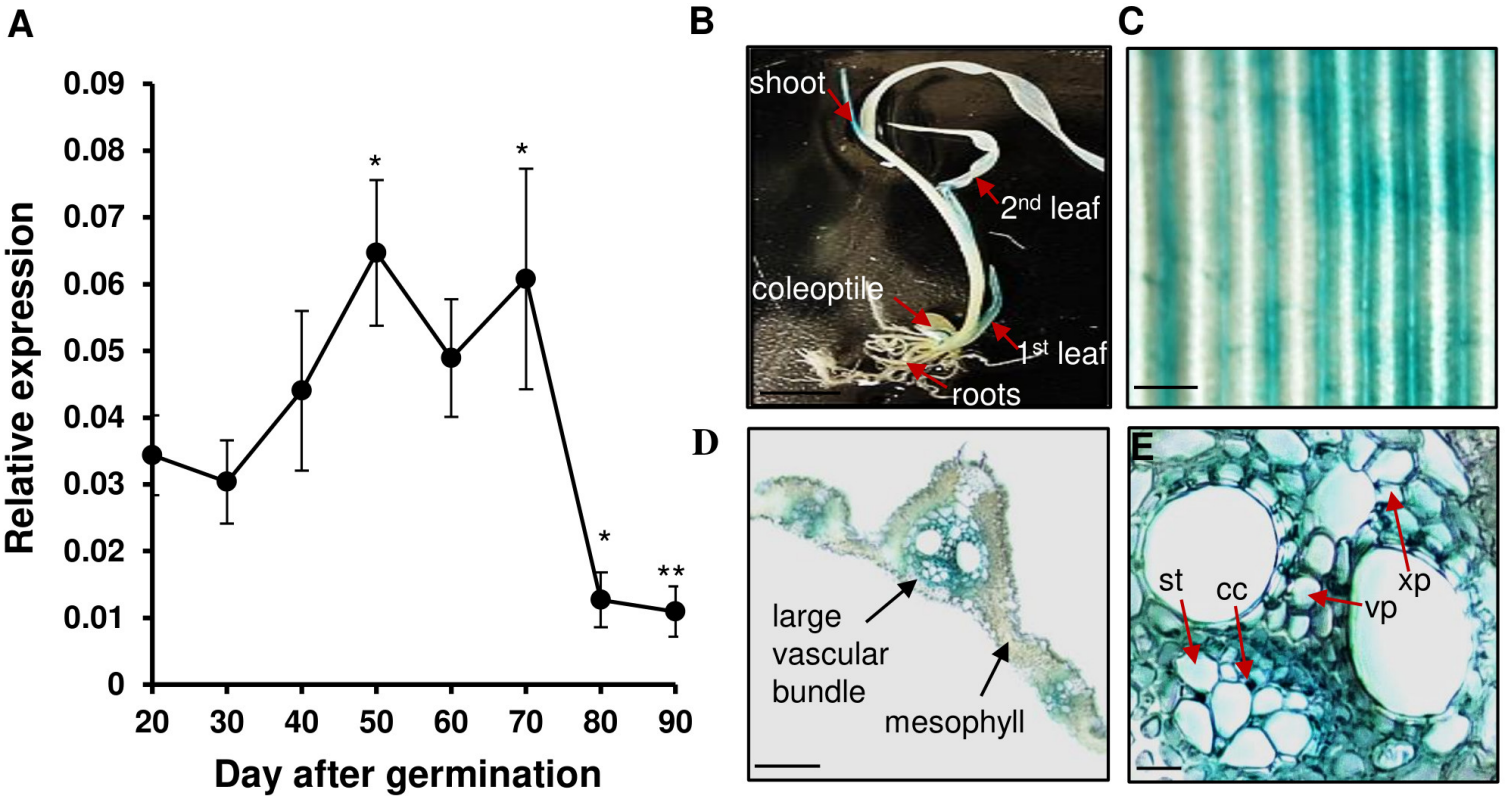
Expression level and GUS signal of *OsWRKY26*. **(A)** The transcript levels of *OsWRKY26* were measured by qRT-PCR. The second leaf from the top was collected at ZT 4 between 20 and 90 DAG. Error bars represent the standard deviation of four replicates. The transcript levels of *OsWRKY26* were normalized to *OsUbi5.* Two-tailed Student’s t-test was used for the statistical analyses, and statistical significance is indicated by * (p < 0.05) ** (p < 0.01). 20 DAG was used as a control to compare with other time points. **(B)** GUS signal of *pOsWRKY26:GUS* seedlings at 10 DAG. Scale bar = 1 cm. **(C)** GUS activity in a leaf blade at 55 DAG. Scale bar = 1 mm. **(D, E)** Cross sections of the leaf blade at 55 DAG. Scale bar = 1 mm (in Figure **(D)**) or 0.2 mm (in Figure **(E)**). cc, companion cells; st, sieve tube; xp, xylem parenchyma; vp, vascular parenchyma.

We previously reported that the expression of *OsWRKY26* in rice Oc cells was induced by supplementation with sucrose within 2 h, reached its highest level after 4 h, and then rapidly declined (Tun et al., 2023). To further characterize the gene, its expression pattern was studied during plant development. The transcript level of *OsWRKY26* gradually increased during the vegetative growth stage and reached the maximum level at 50 DAG (**Figure 1A**). The gene remained highly expressed until 70 DAG, after which transcript levels dropped rapidly at the heading stage (80-90 DAG).

To investigate the tissue-specific expression pattern of *OsWRKY26*, a 3,000 bp sequence upstream of the start codon of *OsWRKY26* was cloned to a *GUS* reporter vector, and the resulting *pOsWRKY26:GUS* vector was transformed into rice plants. GUS staining of tissues from transgenic plants at various developmental stages showed that this signal was detected mainly in leaf vascular bundles (**Figures 1B–E**). In addition, weak GUS staining was found in spikelets (**Supplementary Figure 1A**). However, the staining was not detected in roots (**Supplementary Figures 1B, C**).

### 3.2 OsWRKY26 mutants show enhanced resistance to *Xoo* infection

**Figure 2:**
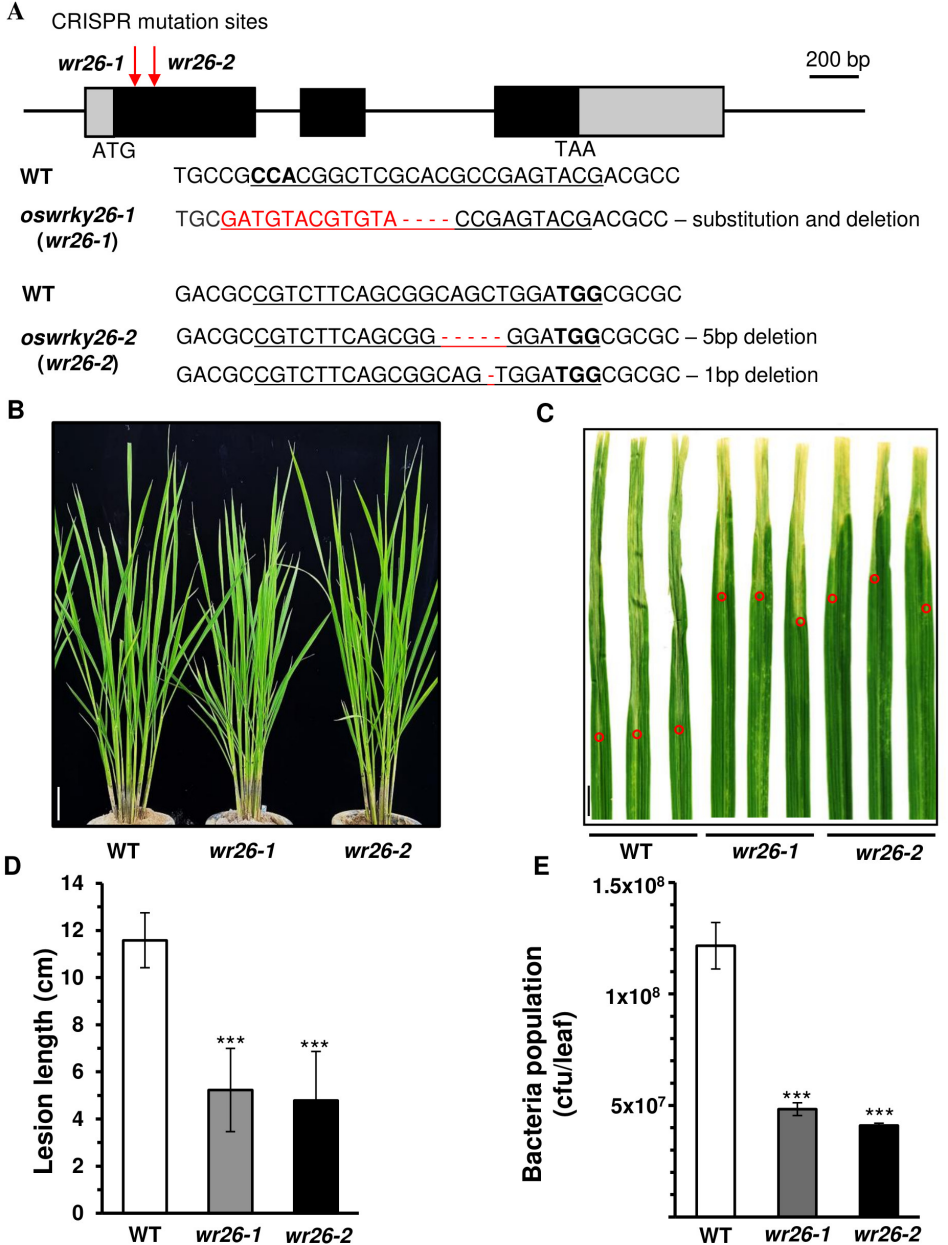
Phenotypes of *oswrky26* mutants created by CRISPR/Cas9 gene editing. **(A)** Schematic diagram of *OsWRKY26* and sequence comparisons of the mutation sites. Gray boxes indicate 5’-UTR and 3’-UTR; black boxes represent exons; lines between boxes represent introns. Red arrows indicate CRISPR target sites. Scale bar = 200 bp. All PAM sites (bold) and target sequences are underlined. Deleted and exchanged sequences in the mutants are highlighted in red. **(B)** Phenotypes of WT, *wr26-1*, and *wr26-2* plants in the T2 generation from paddy fields at 50 DAG. Scale bar = 10 cm. **(C)** Lesions developed by 14 d after infection with *Xoo* strain PXO99. Leaves of WT and mutant plants at 50 DAG were used for *Xoo* inoculation. Scale bar = 1 cm. Red open circles indicate the end of lesion area in each leaf. **(D)** Lesion lengths at 14 d after infection. Error bars represent standard deviation; n = 20. **(E)** *Xoo* population at 12 d after infection. Three infected leaves were used to generate bacterial population counts. Error bars represent the standard deviation of three replicates. Two-tailed Student’s *t*-test was used for all statistical analyses, and statistical significance is indicated by *** (*p <* 0.001).

To investigate the functional role of *OsWRKY26*, null mutants of the gene were generated by the CRISPR/Cas9 methods. Among several transgenic plants, two mutant lines were selected for further study (**Figure 2A**). In the first mutant line, *oswrky26-1* (*wr26-1*), 4 nucleotides were deleted along with a 12-nucleotide substitution at the sgRNA binding site. The second mutant line, *oswrky26-2* (*wr26-2*), carried biallelic mutations: a five base pair deletion in the target site on one chromosome and a one base pair deletion on another chromosome. Both mutant lines were found to grow and show normal head morphology (**Figure 2B**). However, plant height and grain yield were slightly reduced in mutant plants compared to the WT control (**Supplementary Figure 2** and **Supplementary Table 2**).

It has been previously reported that *OsWRKY26* expression can be induced by *Xoo* and *M. oryzae* infection (Choi et al., 2017; Li et al., 2021). We therefore examined whether these mutant lines showed altered responses to *Xoo* infection. Bacterial inoculation of leaves during the juvenile stage (30 DAG) resulted in slightly reduced lesion lengths in mutant lines compared to WT (**Supplementary Figures 3A, B**), with no significant differences observed in heading stage plants (90 DAG) (**Supplementary Figures 3C, D**). However, significant levels of resistance were found in mutant plants at the adult vegetative stage (50 DAG), as evidenced by shorter lesion lengths (**Figures 2C, D**). The bacterial populations were also reduced in the lesions of *wr26* lines compared to the WT (**Figure 2E**). Taken together, these results suggest that enhanced resistance to *Xoo* in the mutants was observed when *OsWRKY26* expression was highest. Notably, no changes in resistance were observed at the heading stage, when *OsWRKY26* expression was low, suggesting that its impact on resistance likely depends on expression levels.

To determine whether *OsWRKY26* also responded to *M. oryzae* infection, we inoculated fungal spores on the second leaf blades of WT, *wr26-1*, and *wr26-2* plants at 50 DAG. Lesion measurements nine days after infection showed similar lesion lengths between WT and mutant plants, suggesting that *OsWRKY26* does not play a significant role in defense against *M. oryzae* (**Supplementary Figures 4A, B**).

### 3.3 Transcriptomic profiling of *oswrky26* mutant suggests induction of defense-related genes

*OsWRKY26* belongs to the WRKY family of TFs, which are known to regulate responses to biotic and abiotic stress, senescence, and various developmental processes (Li et al., 2021; Wang et al., 2023; Javed and Gao, 2023). To confirm the subcellular localization of OsWRKY26, GFP signals from OsWRKY26-GFP and RFP from a nucleus localization signal (NLS-RFP) marker were tracked in protoplasts derived from Oc cells. As a control, the *NLS* was connected to *RFP* under the *ZmUbi1* promoter (NLS-RFP). Both constructs were co-introduced into rice protoplasts and transiently expressed for 14 h. Visualization of the expressed proteins under a fluorescence microscope showed that the OsWRKY26-GFP protein co-localized with NLS-RFP within the nucleus, indicating that OsWRKY26 is a nuclear-localized protein (**Supplementary Figure 5**).

**Figure 3:**
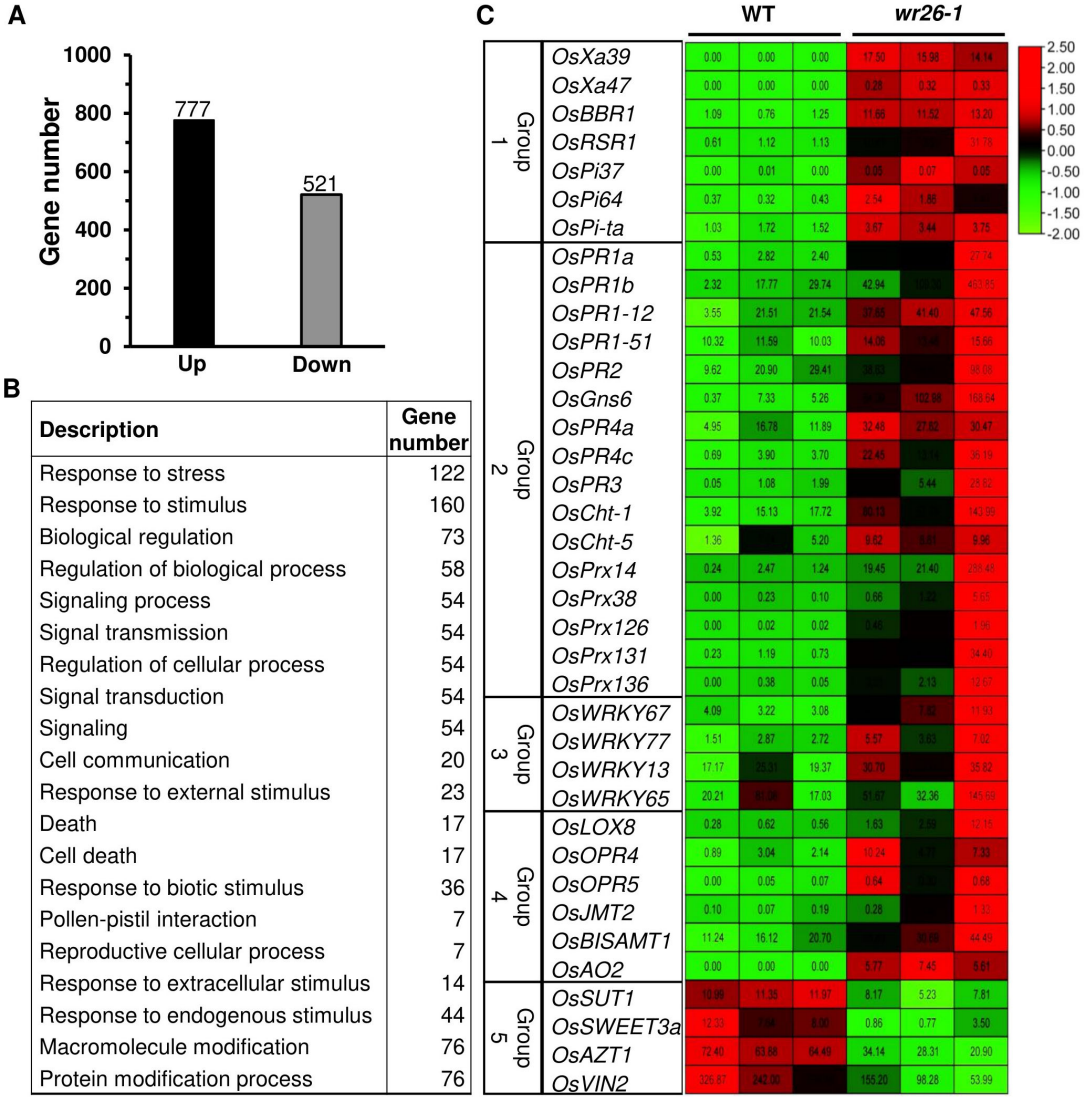
Transcriptomic analyses of three replicates of WT and *wr26* plants. **(A)** Number of up- and downregulated genes in mutant plants. **(B)** Gene Ontology analysis of the biological processes associated with upregulated genes in mutant plants. GO terms are arranged by corrected *p <*0.05. **(C)** Heatmap of disease-related and sugar allocation genes. Numbers within boxes show FPKM values. Red indicates upregulated genes and green indicates downregulated genes.

To investigate the potential targets of *OsWRKY26*, we performed transcriptome analyses of *wr26-1* and WT leaves at the adult vegetative stage. Analysis of three biological replicates revealed 1,298 differentially expressed transcripts between mutant and WT plants. Among them, 777 transcripts were upregulated and 521 were downregulated in the mutant plants (**Figure 3A**, **Supplementary Table 3**). GO analysis revealed that stress- and stimulus-related transcripts were among the most abundantly induced categories in the mutants (**Figure 3B**, **Supplementary Table 4**). Based on their annotated functions, we focused on five groups: Group 1 - NLR genes, Group 2 - PR genes, Group 3 - *WRKY* genes, Group 4 - hormone-related genes, and Group 5 - sugar allocation genes (**Figure 3C**).

**Figure 4:**
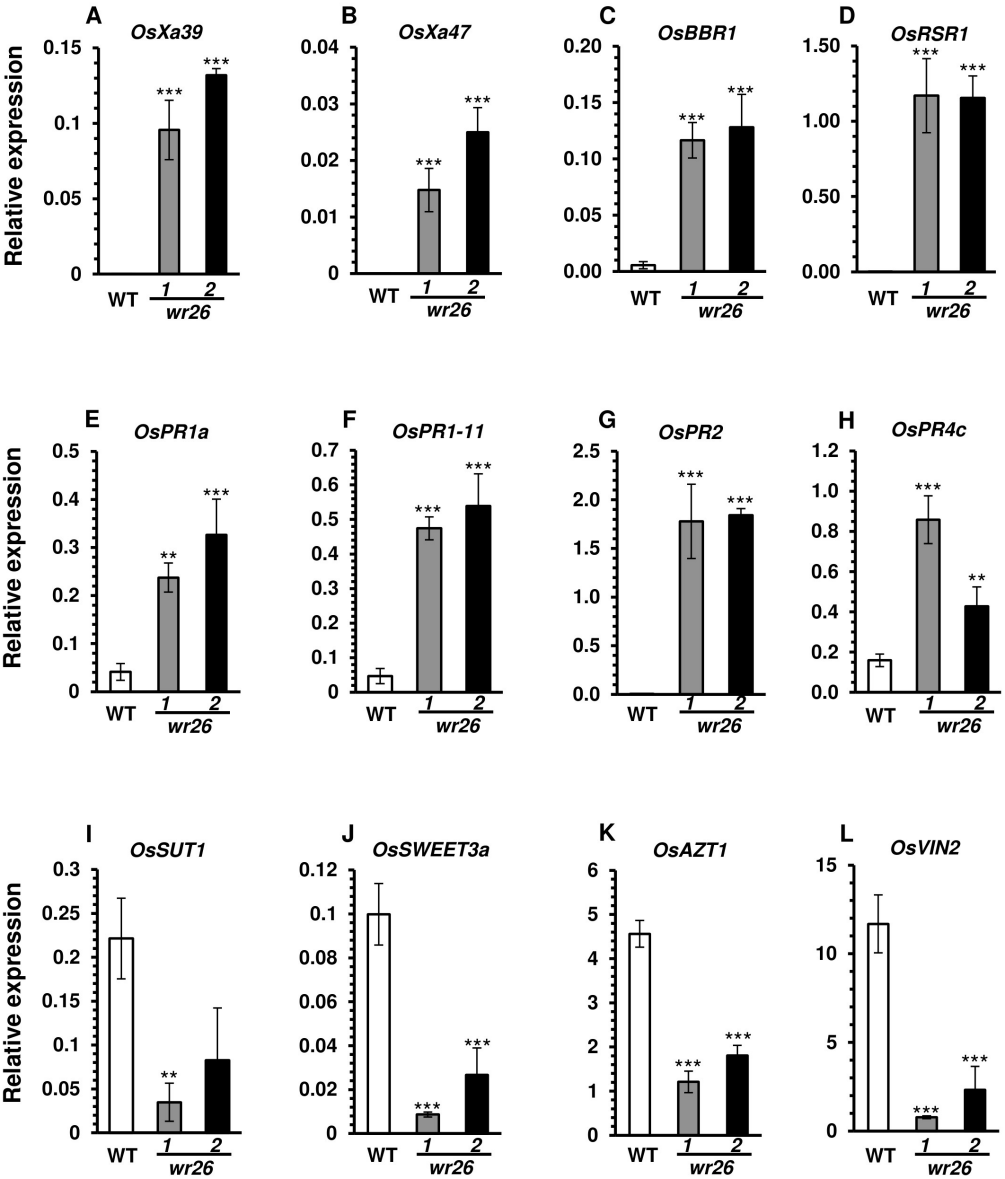
Validation of transcriptomic analyses. **(A–D)** NLR domain-containing genes. **(E–H)** Pathogen-related genes. **(I–L)** Sugar allocation genes. Leaf blades of WT, *wr26-1*, and *wr26-2* plants were collected at 50 DAG. RNA concentrations were normalized to those of *OsUbi5*. Errors bars represent standard deviation. n = 4. Statistical significance is indicated by ** (*p <*0.01) and *** (*p <* 0.001).

Since *wr26* mutant plants showed enhanced resistance to bacterial pathogen infection, we chose eight genes from Group 1 and Group 2 that were related to disease resistance for further analysis. The expression levels of several NLR genes, *OsXa39*, *OsXa47*, *OsBBR1*, and *OsRSR1*, and PR genes, *OsPR1a*, *OsPR1b*, *OsPR2*, and *OsPR4c*, were significantly higher in *wr26* mutant leaves compared to the WT (**Figures 4A–H**). In contrast, RNA-seq data revealed that transcript levels of genes involved in sugar allocation were significantly lower in mutant plants compared to the WT (**Figures 4I–L**). These results indicate that *OsWRKY26* plays a significant role in regulating defense-related genes, thereby enhancing resistance to *Xoo* infection, while also affecting sugar metabolism pathways.

### 3.4 Identification of OsWRKY26 repressor region on the *OsXa39* promoter

**Figure 5:**
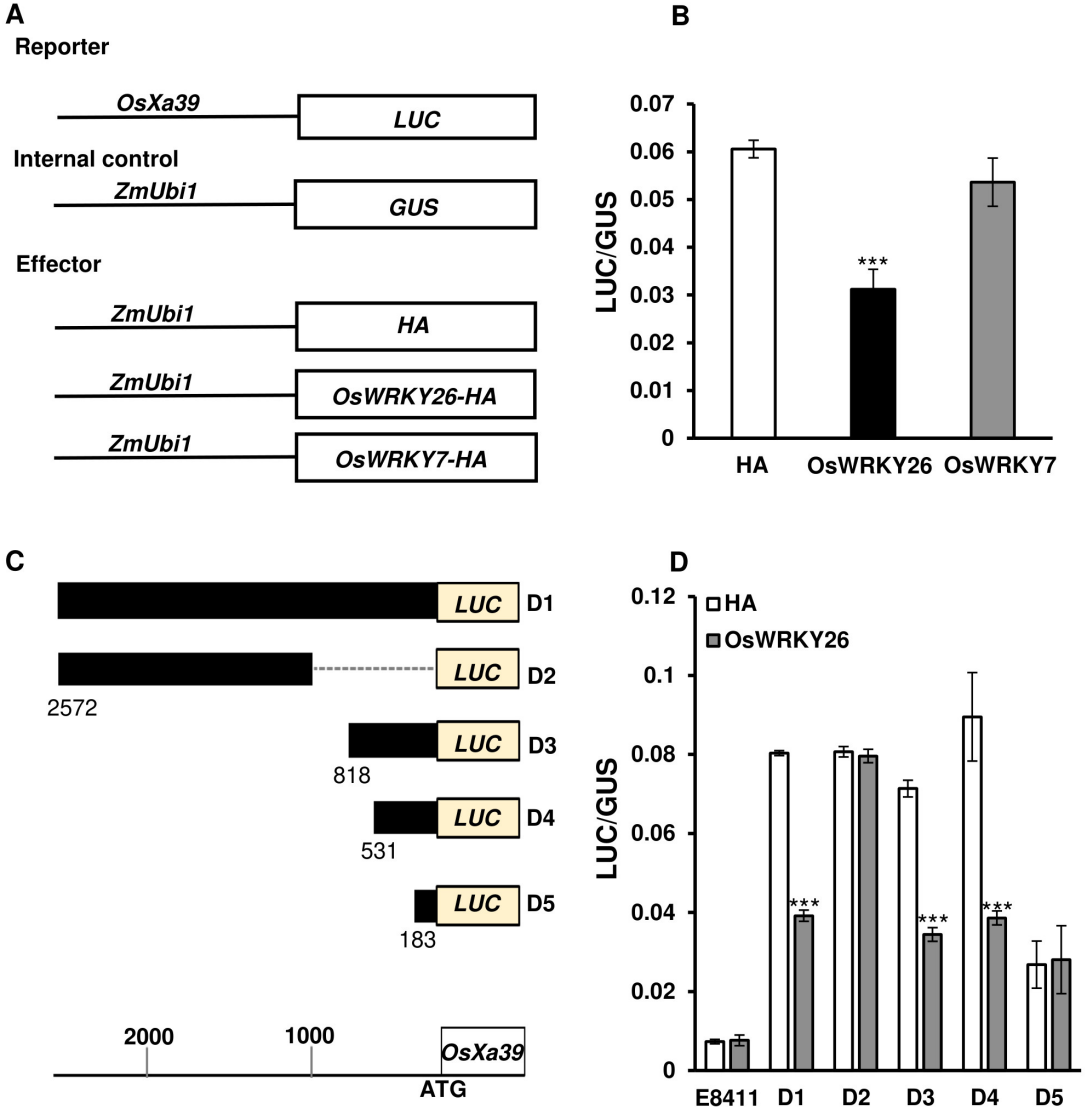
Transient expression analyses of the *OsXa39* promoter. **(A)** Schematic diagrams of *OsXa39* reporter and effector constructs. **(B)** Luciferase activity driven by the *OsXa39* promoter, normalized to GUS activity driven by the *ZmUbi1* promoter. Effector constructs, *HA*, *OsWRKY26*, or *OsWRKY7* were connected to the *ZmUbi1* promoter then co-introduced to Oc cell protoplasts alongside reporter constructs. Error bars represent standard deviation; n = 3. **(C)** Schematic diagrams of reporter constructs showing different regions of the *OsXa39* promoter and *Luciferase* (*LUC*) gene. D1, 2,572 to 63 bp; D2, 2,572 to 1,022 bp; D3, 818 to 63 bp; D4, 531 to 63 bp; D5, 183 to 63 bp upstream of the ATG start codon. Scale bar = 200 bp. **(D)** Luciferase assay used to identify the repressive binding region of OsWRKY26 to the *OsXa39* promoter. *ZmUbi : GUS* was used as an internal control. LUC/GUS represents the ratio of luciferase activity driven by the *OsXa39* promoter to the GUS activity of the control. Error bars represent standard deviation; n = 4. E8411 (pGL4.23) is the original reporter vector. Experiments were repeated three times. Statistical significance is indicated by *** (*p* < 0.001).

As NLR genes are preferentially respond to *Xoo* strains (Lu et al., 2022), we focused on the Group I from **Figure 1C**. Because *OsXa39* confers broad-spectrum resistance to various *Xoo* strains compared with other NLR genes (Shi et al., 2022; Zhang et al., 2015a, 2015b; Zhang et al., 2015c), we investigated whether this gene is a direct target of OsWRKY26 by employing a transient expression assay. The *OsXa39* promoter region (−2,572 to −63 bp upstream of the start ATG codon) was fused to the *Luciferase* gene (**Figure 5A**). As a reference, the *GUS* reporter was driven by the *ZmUbi1* promoter. Both constructs were co-transformed into rice protoplasts along with an OsWRKY26-HA fusion protein-expressing plasmid (**Figure 5A**). As a control, a plasmid expressing only the HA tag was also introduced. After 15 h of incubation, the luciferase and GUS activities were measured. The results showed that luciferase activity driven by the *OsXa39* promoter was significantly reduced when co-expressed with *OsWRKY26* compared to the HA alone (**Figure 5B**). As a further control, we introduced *OsWRKY7*, a gene involved in resistance to both *M. oryzae* and *Xoo*, and found that this did not affect *OsXa39* expression (**Figure 5B**). These results indicated that *OsXa39* promoter activity was specifically repressed by OsWRKY26.

To locate the region responsible for the repression, two constructs were generated: one containing an upstream region (−2,572 to −1,022 bp) and the other containing a downstream region (−818 to −63 bp) (**Figure 5C**). These regions were connected to the *Luciferase* gene and introduced into rice protoplasts along with OsWRKY26-HA or with only the HA tag. Measurement of luciferase activity showed that expression of *Luciferase* driven by the upstream region was not inhibited by OsWRKY26 (**Figure 5D**). However, the marker expression linked to the downstream region was significantly suppressed by OsWRKY26 (**Figure 5D**). To further narrow down the repressor region, two additional fragments (−531 to −63 bp and −183 to −63 bp) were tested (**Figure 5C**). Transient assay results revealed that the longer fragment retained the repressor activity, whereas the shorter fragment did not (**Figure 5D**). Therefore, the OsWRKY26 repressor region is likely located between −531 and −183 bp.

OsWRKY26 may bind to and interfere with an unspecified positive regulatory transcription factor that induces expression of *OsXa39*. To find potential TFs interacting with OsWRKY26, we conducted a yeast two-hybrid screen, which identified 19 interacting proteins, including E3 ligases, enzymes, hormone-related genes, and other proteins of unknown function. However, no TFs known to be involved in *Xoo* resistance were identified (**Supplementary Table 5**). These results suggest that OsWRKY26 may directly bind to the promoter region of *OsXa39* and repress its expression.

To confirm whether OsWRKY26 directly binds to the *OsXa39* promoter, we performed ChIP-PCR analysis. Overexpression lines of *OsWRKY26* were generated using a construct containing the *OsWRKY26* coding region between *ZmUbi1* promoter and a Myc tag (**Supplementary Figure 6A**). Leaf samples from six transgenic plants were harvested to measure OsWRKY26-Myc protein levels by western blot analysis (**Supplementary Figure 6B**). Three transgenic plants expressed the fusion protein at high levels, and the transcript levels of lines #9 and #10 were further confirmed (**Supplementary Figure 6C**). These transgenic plants did not show altered responses to *Xoo* (**Supplementary Figures 6D, E**).

**Figure 6:**
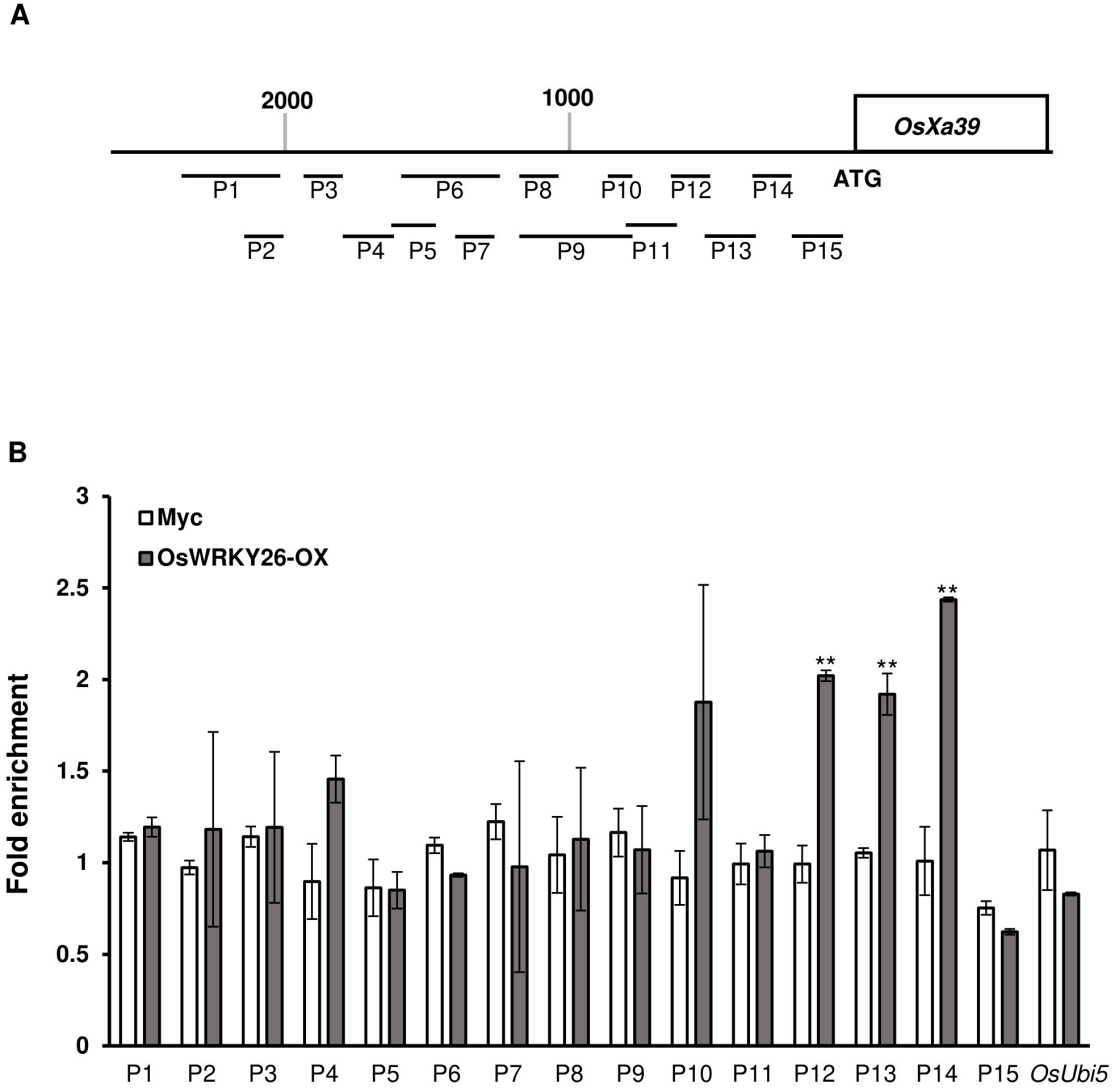
Chromatin immunoprecipitation assay of the promoter region of *OsXa39.* **(A)** Schematic diagram of the *OsXa39* promoter and amplicon locations. Fifteen amplicons and *OsUbi5* were used for this analysis: P1, 2,414 to 2,017 bp; P2, 2,163 to 2,017 bp; P3,1,906 to 1,782 bp; P4,1,805 to 1,611 bp; P5, 1,631 to 1,481 bp; P6, 1,631 to 1,236 bp; P7, 1,372 to 1,236 bp; P8, 1,164 to 1,022 bp, P9, 1,164 to 818 bp; P10, 914 to 818 bp; P11, 840 to 658 bp; P12, 680 to 531 bp; P13, 554 to 324 bp; P14 348 to 191 bp; and P15 201 to 1 bp upstream of the ATG start codon. Scale bar = 200 bp. **(B)** Fold enrichment of OsWRKY26 binding to fifteen regions of the *OsXa39* promoter. Chromatins from *OsWRKY26-OX* plants at 10 DAG was immunoprecipitated with Myc antibody, and the enriched DNA was quantified by qRT-PCR. Error bars represent standard deviation; n = 2. Experiments were repeated three times. Statistical significance is indicated by ** (*p* < 0.01).

Transgenic plants expressing *OsWRKY26* at high levels (OsWRKY26-OX*-*9) and those expressing only the Myc tag were used for ChIP-PCR. Chromatins from transgenic nuclei were sonicated and immunoprecipitated with Myc antibody. Precipitated DNA was amplified with fifteen overlapping primer sets spanning 96-397 bp of the promoter region of *OsXa39* (**Figure 6A**). In these tests, *OsUbi5* was used as a negative control to test for non-specific binding of the antibody. The analysis showed that three regions, P12 (−680 to −531 bp), P13 (−554 to −324 bp), and P14 (−348 to −191 bp), were enriched with OsWRKY26, indicating that OsWRKY26 binds directly to the *OsXa39* promoter region between −680 and −191 bp upstream of the start codon (**Figure 6B**). We also noted that this region overlapped with the OsWRKY26 repressor region (−531 and −183 bp) identified by the transient expression assay using the *Luciferase* marker. These results highlight the repressive activity of OsWRKY26 on the *OsXa39* promoter.

## 4 Discussion

WRKY TFs are pivotal regulators of plant defense mechanisms, modulating responses against various biotic stresses. Their functional diversity enables plants to mount appropriate responses to different pathogens with distinct infection strategies. WRKY proteins can act as both positive and negative regulators of defense, depending on the pathogen and context. For example, *OsWRKY7* and *OsWRKY67* are known to induce PR gene expression, which enhances defense against *Xoo* and *M. oryzae* (Liu et al., 2018; Tun et al., 2023; Vo et al., 2018; Zhao et al., 2024; Zheng et al., 2023). On the other hand, several WRKY proteins act as negative regulators of defense. For example, *OsWRKY62* represses defense pathways, leading to increased vulnerability to *Xoo* (Peng et al., 2008).

We found that *OsWRKY26* is preferentially expressed in vascular bundles, and its expression pattern during plant development correlates with key growth stages, peaking at 50 DAG. Importantly, *OsWRKY26* expression is rapidly induced by sucrose supplementation and remains high during vegetative growth, suggesting a functional link between sugar signaling and defense response. This finding aligns with the function of *OsWRKY26* in regulating defense against vascular-targeting pathogens like *Xoo*.

Several *WRKY* genes exhibit multifunctionality, playing roles in defense against different biotic stressors. For example, *OsWRKY45* is a gene with dual functions: its ectopic expression enhances resistance to both *Xoo* and *M. oryzae*, while loss-of-function mutations confer resistance against the necrotrophic pathogen *Rhizoctonia solani* and the piercing-sucking insect *Nilaparvata lugens* (Shimono et al., 2012; Huangfu et al., 2016). Interestingly, some defensive responses involve tradeoffs; for example, overexpression of *OsWRKY70* enhances resistance to the chewing herbivore *Chilo suppressalis* as well as increased susceptibility to the brown planthopper *N. lugens* (Li et al., 2015). This study revealed that *OsWRKY70* promotes resistance to *C. suppressalis* by enhancing JA biosynthesis and suppressing GA signaling, while its GA-dependent regulation increases susceptibility to *N. lugens*, highlighting the tradeoff between defense and growth. These examples highlight the flexibility of *WRKY* genes like *OsWRKY45* and *OsWRKY70* in modulating different defense pathways. Similarly, *OsWRKY53* has been found to affect both bacterial and fungal resistance. For example, mutations in *OsWRKY53* lead to thickened sclerenchyma cell walls and increased resistance to *Xoo*, while its overexpression enhances resistance to *M. oryzae* through activation of PR genes (Chujo et al., 2007, 2014; Xie et al., 2021). This suggests that *OsWRKY53* can regulate both structural defenses and defense signaling pathways. Previously, we mentioned that *OsWRKY26* were strongly induced by sugars. Mutation of *OsWRKY26* increased resistance to *Xoo* infection and up-regulated the expression level of several defense related genes, on the other hands, reduced some sugar related genes, suggesting that *OsWRKY26* might control the trade-offs mechanisms between defense mechanisms and sugar allocation during the plant development.

Our findings indicate that while *OsWRKY26* is induced by both *Xoo* and *M. oryzae* (Choi et al., 2017; Li et al., 2021; Tun et al., 2023), it plays a distinct role specifically in defense against *Xoo*. This pathogen-specific regulation may reflect differences in infection strategies employed by these two pathogens. *Xoo*, as an obligate biotroph, primarily targets vascular tissues, while *M. oryzae* transitions from a biotrophic phase to necrotrophy during infection. The lack of enhanced resistance to *M. oryzae* in *wr26* mutants suggests that *OsWRKY26* is more closely linked to vascular-based defense, which are crucial for combating *Xoo*.

Transcriptome analysis of *wr26* mutants revealed that the loss of *OsWRKY26* function leads to the upregulation of various genes linked to resistance, including NLRs and PR genes. For example, among NLR genes, *OsXa39* is a gene that is known to confer resistance to 21 *Xoo* strains via hypersensitive response mechanisms (Zhang et al., 2015c). Moreover, *OsXa47* is involved in *Xoo* resistance (Lu et al., 2022), with overexpression of this gene causing the induction of *OsNPR1*, *OsPR1a*, and *OsPR10a.* In addition, knockout mutations of *OsXa47* has been found to lead to the downregulation of PR genes. Overexpression of *OsBBR1* has been previously found to moderately increase resistance to the *Xoo* strains PXO86 and PXO341 (Wang et al., 2013), and *OsRSR1* was found to contribute to sheath blight resistance by ROS modulation and antioxidation activity in rice (Wang et al., 2021).

Several PR genes were also significantly induced in *wr26* mutants. These include *OsPR1a*, which is induced by infection by *Xoo* as well as by the fungal strains, *M. oryzae* and *Rhizoctonia solani*. RNA silencing of *OsPR1a* has been found to result in longer lesion lengths (Yan et al., 2022), whereas its overexpression shortens lesion length (Liu et al., 2021). Overexpression of another gene, *OsPR1-11* (also known as *OsPR1b*), has been found to strengthen resistance to *Xoo* (Luan and Zhou, 2015). More generally, the OsPR2 protein, which encodes a β- 1,3-glucanase, is known to be involved in pathogen defense response, where it degrades fungal cell wall components (Kaur et al., 2017), and *OsPR4c*, part of the PR4 family, is induced by *M. oryzae* infection and has several antifungal properties (Wang et al., 2011). This finding is also consistent with previous reports, which found that WRKY TFs regulate these types of defense genes (Javed and Gao, 2023). One notable finding is the upregulation of *OsXa39*, a broad-spectrum resistance gene that confers resistance to multiple *Xoo* strains (Zhang et al., 2015c). The strong upregulation of *OsXa39* in *wr26* mutants suggests that *OsWRKY26* functions as a repressor of this gene, and can fine-tune its expression under normal conditions to prevent excessive defense activation. This regulation is important because excessive or untimely defense activation can lead to unnecessary energy expenditures, which affect overall plant growth and fitness (Huot et al., 2014). Further studies on other NLR and PR genes regulated by *OsWRKY26* will provide deeper insights into its role in disease resistance.

It was recently reported that the TGAC(N)TGAC sequence is bound by WRKY transcription factors with higher efficiency compared to the TGAC(N)ACGT sequence. While the ACGT motif is activated under stress conditions, the TGAC motif is generally downregulated. This suggests that the TGAC motif may serve as a binding site for negative regulators or repressors (Dhatterwal et al., 2019). Molecular analyses, including ChIP-PCR and promoter deletion assays, identified a repressor-binding region located between −531 and −183 bp upstream of the *OsXa39* start codon. This region contains three TGAC motifs, which have been associated with repressor binding under non-biotic stress conditions (Dhatterwal et al., 2019). Our findings suggest that OsWRKY26 binds directly to these motifs to suppress *OsXa39* expression. This repressive regulation likely plays a critical role in balancing host defense and growth, thereby enabling the plant to conserve energy during non-infectious conditions.

Interestingly, our data also suggest that *OsWRKY26* regulates sugar allocation in vascular tissues. Several genes involved in sucrose transport and allocation, such as *SUT1*, *VIN2*, *SWEET3a*, and *TMT2*, were downregulated in *wr26* mutants. *Xoo* is known to hijack plant sugar transport pathways by activating *SWEET* genes through TAL effectors. TAL proteins contain key domains: a type III secretion signal for host cell entry, a central repeat region binding to effector binding elements in *SWEET* gene promoters, a nuclear localization signal, and an activation domain. As sucrose cannot passively diffuse through the plasma membrane, SWEET proteins mediate its translocation to the apoplasm of phloem cells, providing nutrients in the cell wall space where *Xoo* propagates (Mondal et al., 2021; Gupta et al., 2021; Verdier et al., 2012). The reduced expression of *SWEET3a* and other sugar transporters in *wr26* mutants may result in a reduction in the resources available for *Xoo* growth, thereby contributing to the enhanced resistance observed in these plants. Overall, our data therefore suggest that *OsWRKY26* plays a dual role in regulating both sugar transport and defense responses.

The slight reduction in plant height and grain yield observed in *wr26* mutants suggests that *OsWRKY26* also plays a role in growth regulation, likely by balancing defense and growth pathways. As many defense-related genes were upregulated in the mutants, it is possible that the activation of these pathways diverts energy and resources away from growth, resulting in the observed phenotype. In general, tradeoffs between defense activation and growth are common in plants, with enhanced defense often coming at the expense of growth and reproductive success (Yang et al., 2023). Alternatively, the lack of *OsWRKY26* reduced sucrose allocation to sink tissues, causing the growth reduction and yield loss. Further study is needed to investigate the role of *OsWRKY26* in plant growth and development.

In conclusion, *OsWRKY26* functions as a negative regulator of defense responses against *Xoo* and plays a crucial role in balancing defense and growth through its regulation of key resistance genes such as *OsXa39* and sugar transport pathways. By fine-tuning immune responses, *OsWRKY26* enables plants to respond effectively to bacterial pathogens while minimizing negative impacts on growth and energy resources. The dual role of *OsWRKY26* in modulating both defense and sugar allocation provides new insights into the complex regulatory networks that govern plant immunity and growth. Further research is required to explore how *OsWRKY26* coordinates these processes by interacting with other signaling pathways. A deeper understanding of these mechanisms could support the development of rice cultivars with enhanced *Xoo* resistance that maintain overall plant fitness.

## Data Availability

The data presented in the study are deposited in the NCBI repository, accession number PRJNA1201887.
