# Supplementary material for: OsWRKY26 negatively regulates bacterial blight resistance by suppressing OsXa39 expression
Source: Front Plant Sci. 2025 Jan 9;15:1519039. doi: 10.3389/fpls.2024.1519039 (PMC11754229; doi:10.3389/fpls.2024.1519039)
Supplement: Supplementary file 2 [file Presentation1.pptx]

## Slide 1
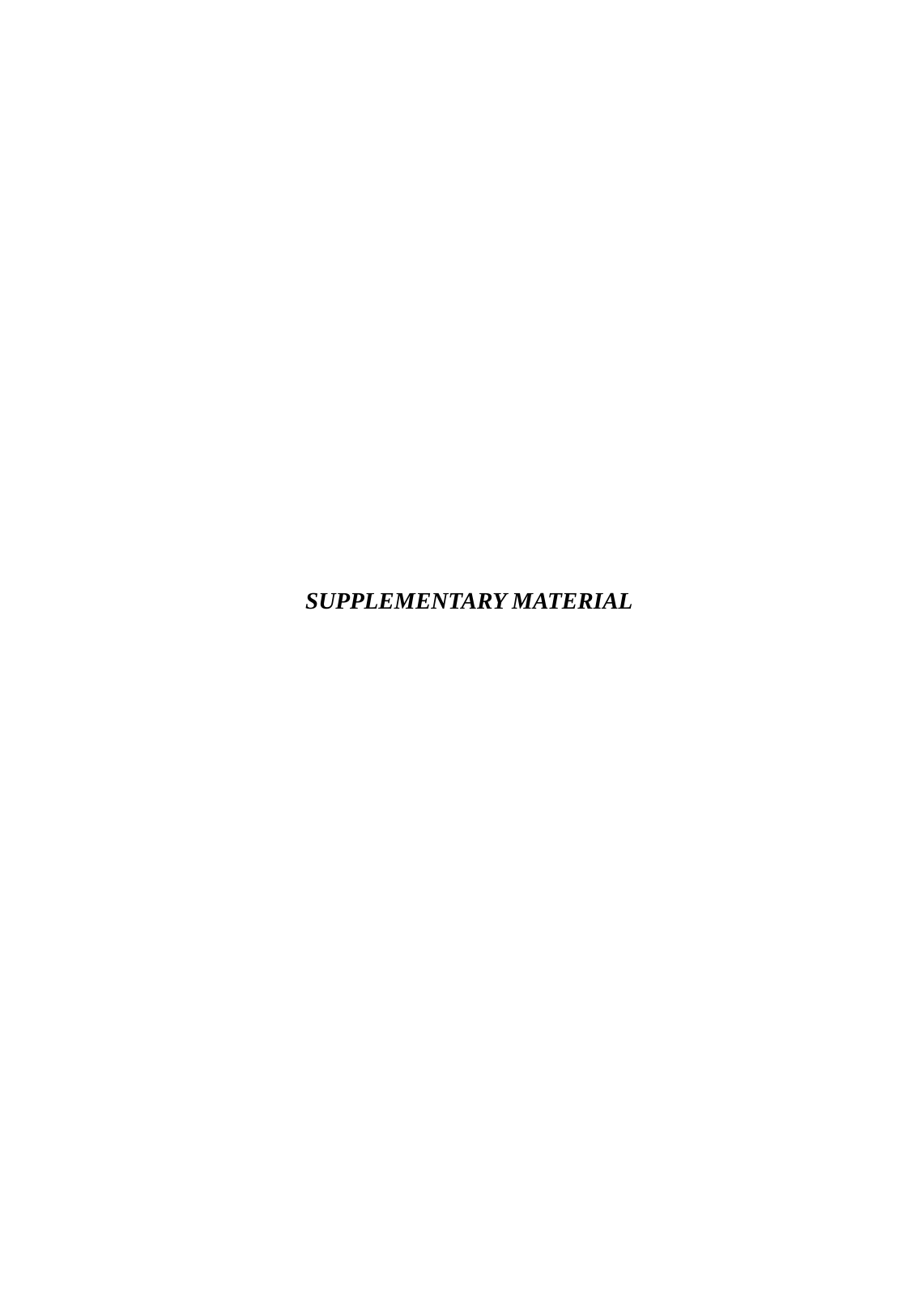

Supplementary Material

## Slide 2
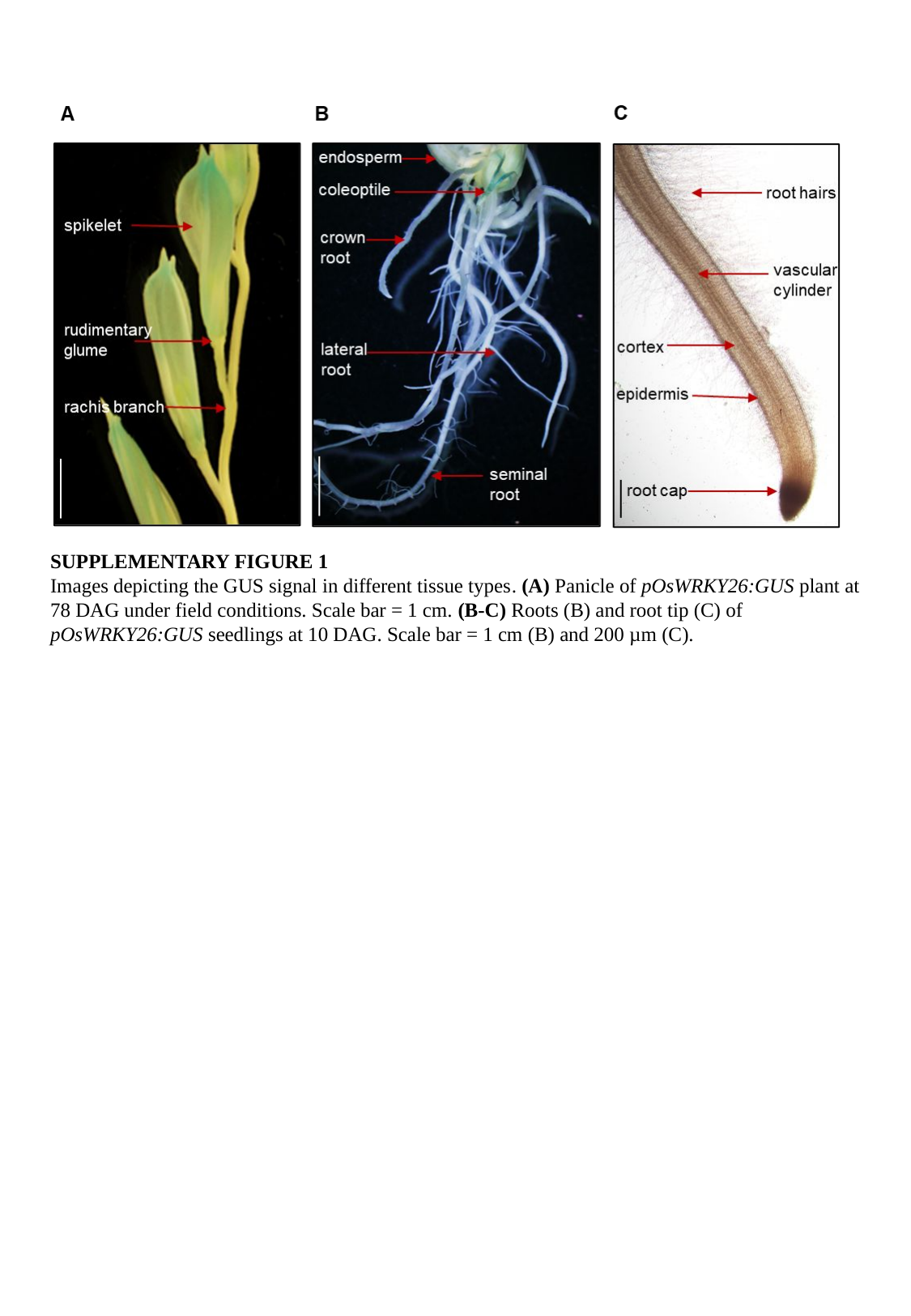

SUPPLEMENTARY FIGURE 1
Images depicting the GUS signal in different tissue types. (A) Panicle of pOsWRKY26:GUS plant at 78 DAG under field conditions. Scale bar = 1 cm. (B-C) Roots (B) and root tip (C) of pOsWRKY26:GUS seedlings at 10 DAG. Scale bar = 1 cm (B) and 200 µm (C).

## Slide 3
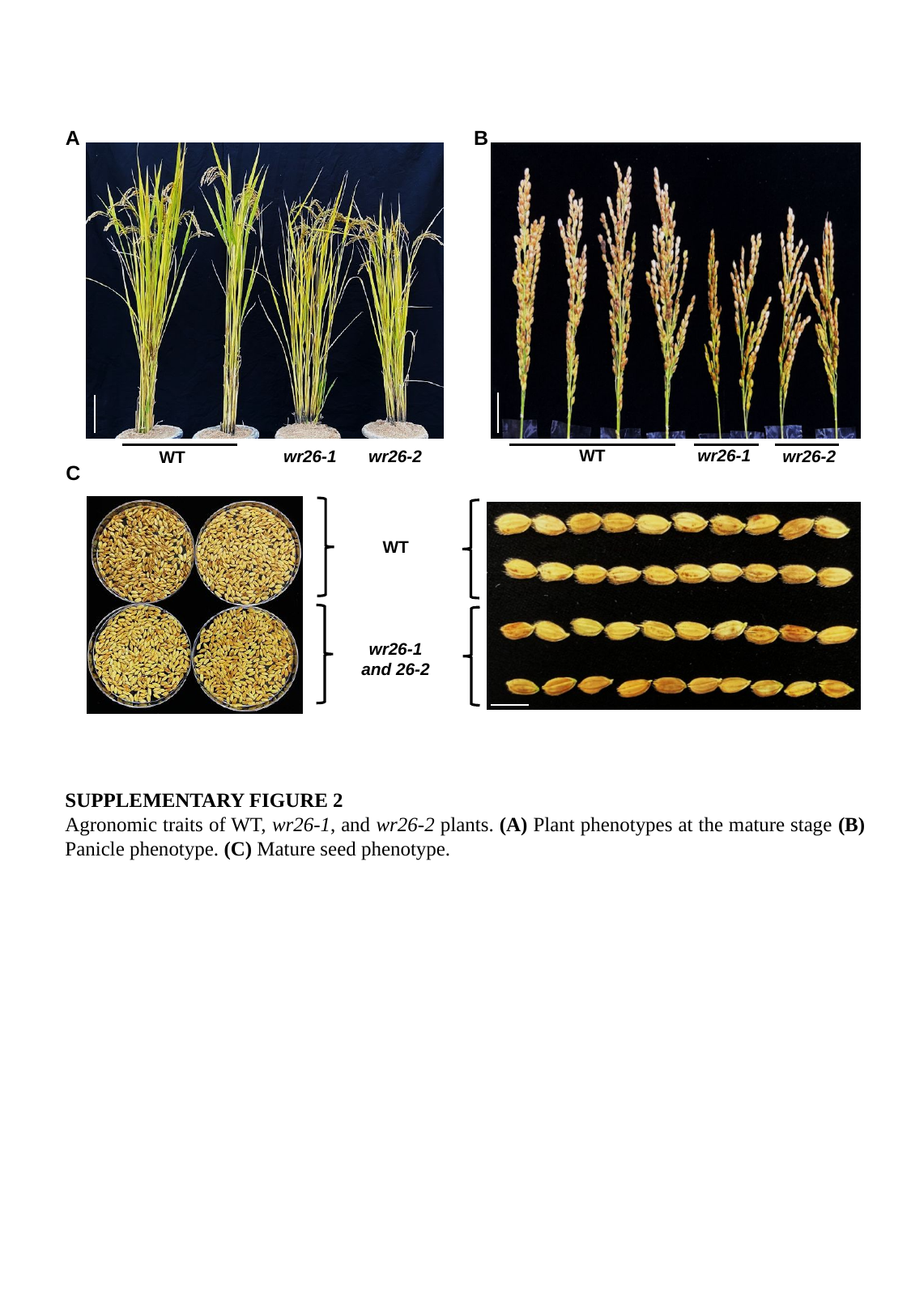

A
B
WT
wr26-1
wr26-2
wr26-2
wr26-1
WT
C
WT
wr26-1
and 26-2
SUPPLEMENTARY FIGURE 2
Agronomic traits of WT, wr26-1, and wr26-2 plants. (A) Plant phenotypes at the mature stage (B) Panicle phenotype. (C) Mature seed phenotype.

## Slide 4
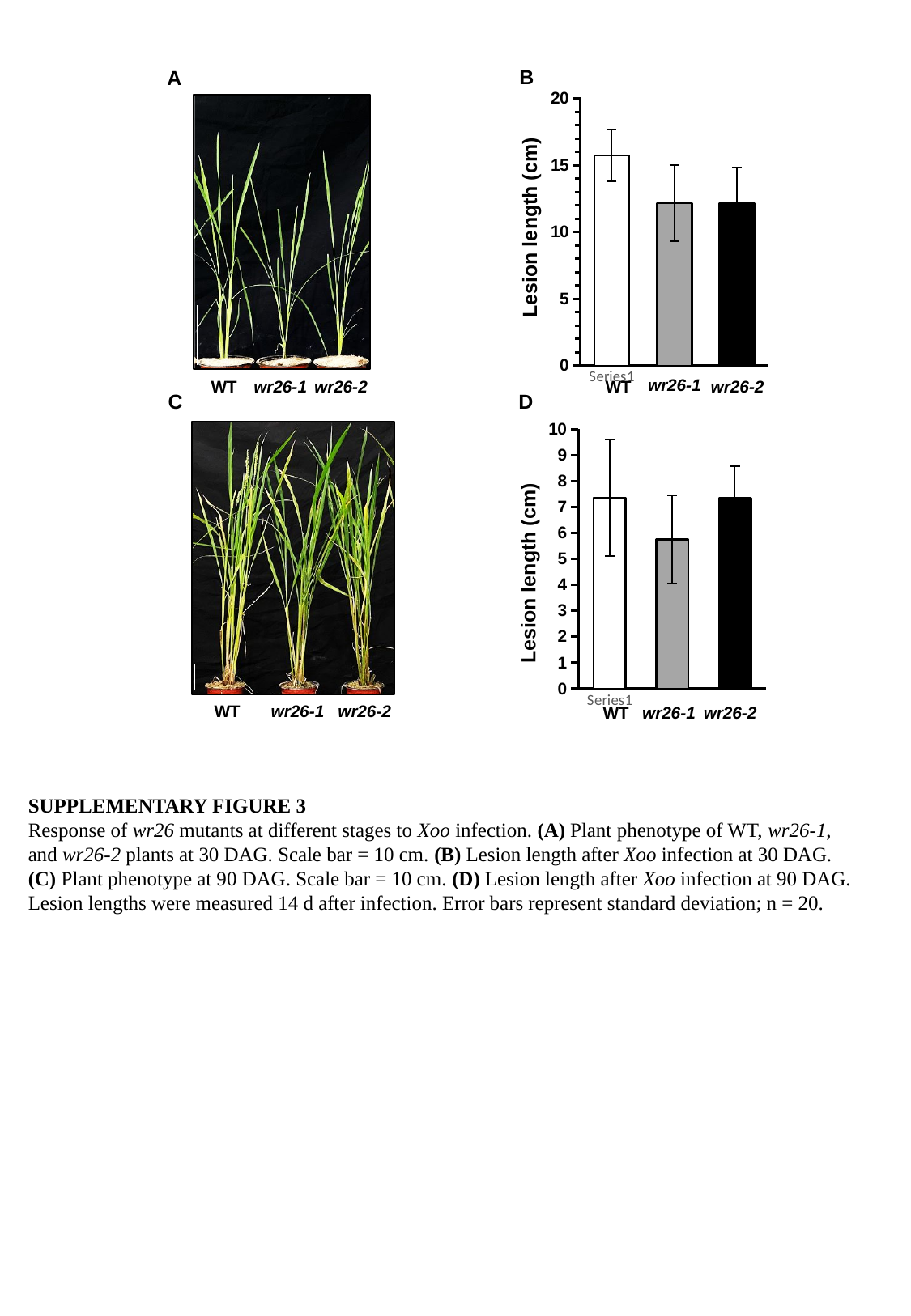

B
A
Lesion length (cm)
wr26-1
WT
wr26-2
### Chart
| Category | |
|---|---|
| | 15.736842105263158 |
| | 12.172413793103448 |
| | 12.172413793103448 |wr26-2
wr26-1
WT
C
D
Lesion length (cm)
wr26-2
WT
wr26-1
### Chart
| Category | |
|---|---|
| | 7.36 |
| | 5.75 |
| | 7.35 |wr26-2
wr26-1
WT
SUPPLEMENTARY FIGURE 3
Response of wr26 mutants at different stages to Xoo infection. (A) Plant phenotype of WT, wr26-1, and wr26-2 plants at 30 DAG. Scale bar = 10 cm. (B) Lesion length after Xoo infection at 30 DAG. (C) Plant phenotype at 90 DAG. Scale bar = 10 cm. (D) Lesion length after Xoo infection at 90 DAG. Lesion lengths were measured 14 d after infection. Error bars represent standard deviation; n = 20.

## Slide 5
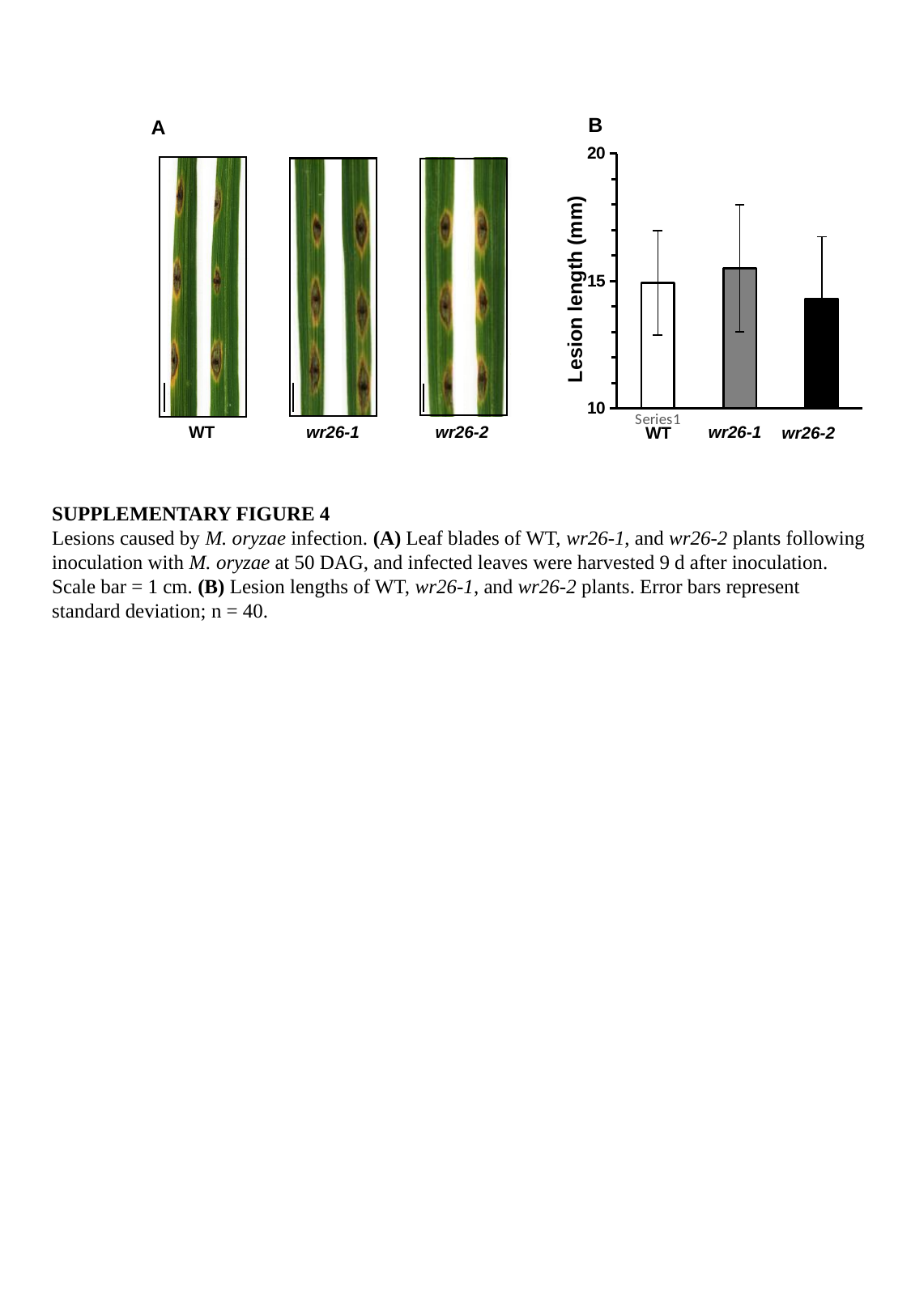

B
A
### Chart
| Category | |
|---|---|
| | 14.931400000000002 |
| | 15.492442307692304 |
| | 14.301024390243905 |
Lesion length (mm)
wr26-1
wr26-2
WT
WT
wr26-1
wr26-2
SUPPLEMENTARY FIGURE 4
Lesions caused by M. oryzae infection. (A) Leaf blades of WT, wr26-1, and wr26-2 plants following inoculation with M. oryzae at 50 DAG, and infected leaves were harvested 9 d after inoculation. Scale bar = 1 cm. (B) Lesion lengths of WT, wr26-1, and wr26-2 plants. Error bars represent standard deviation; n = 40.

## Slide 6
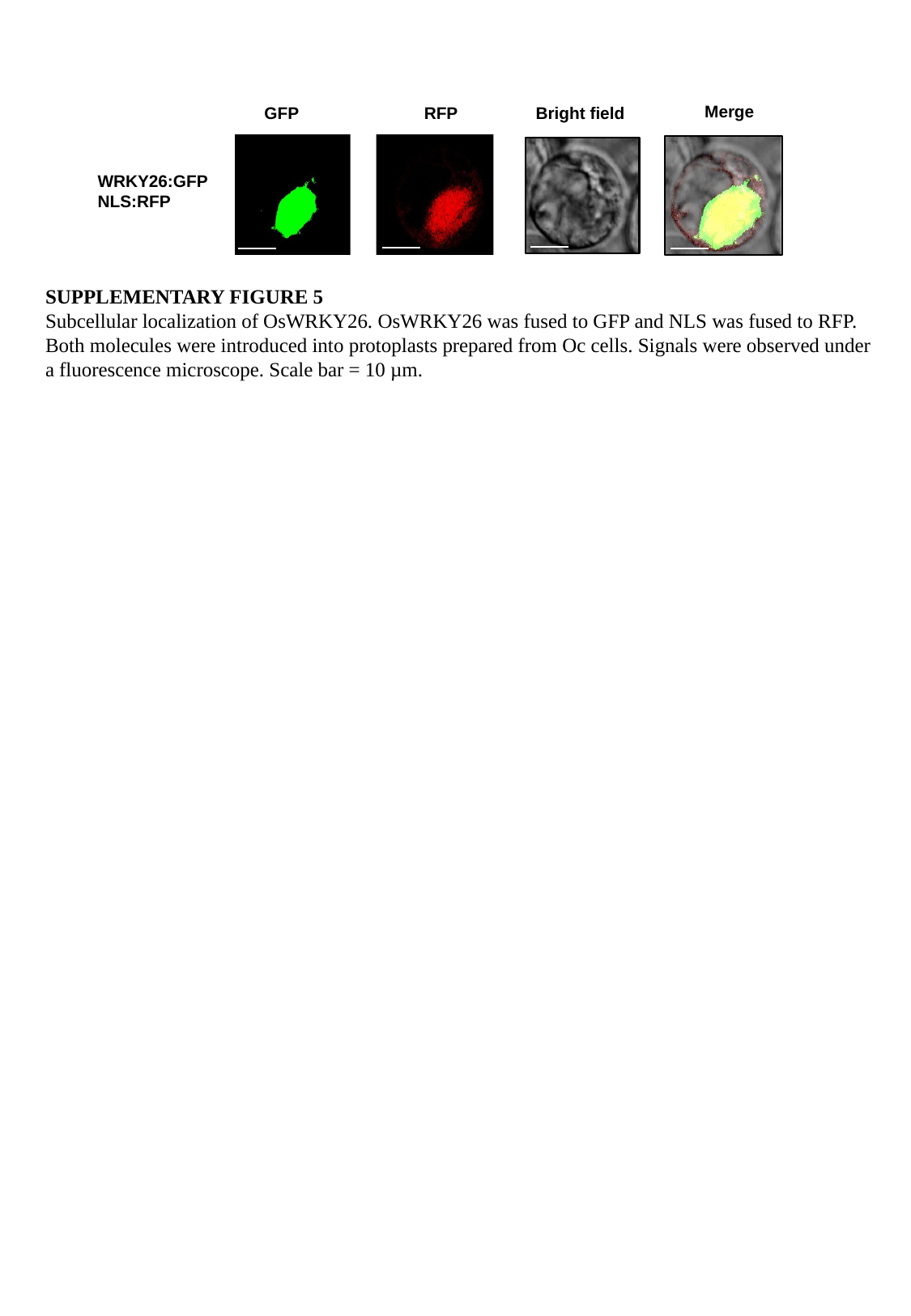

Merge
Bright field
RFP
GFP
WRKY26:GFP
NLS:RFP
SUPPLEMENTARY FIGURE 5
Subcellular localization of OsWRKY26. OsWRKY26 was fused to GFP and NLS was fused to RFP. Both molecules were introduced into protoplasts prepared from Oc cells. Signals were observed under a fluorescence microscope. Scale bar = 10 µm.

## Slide 7
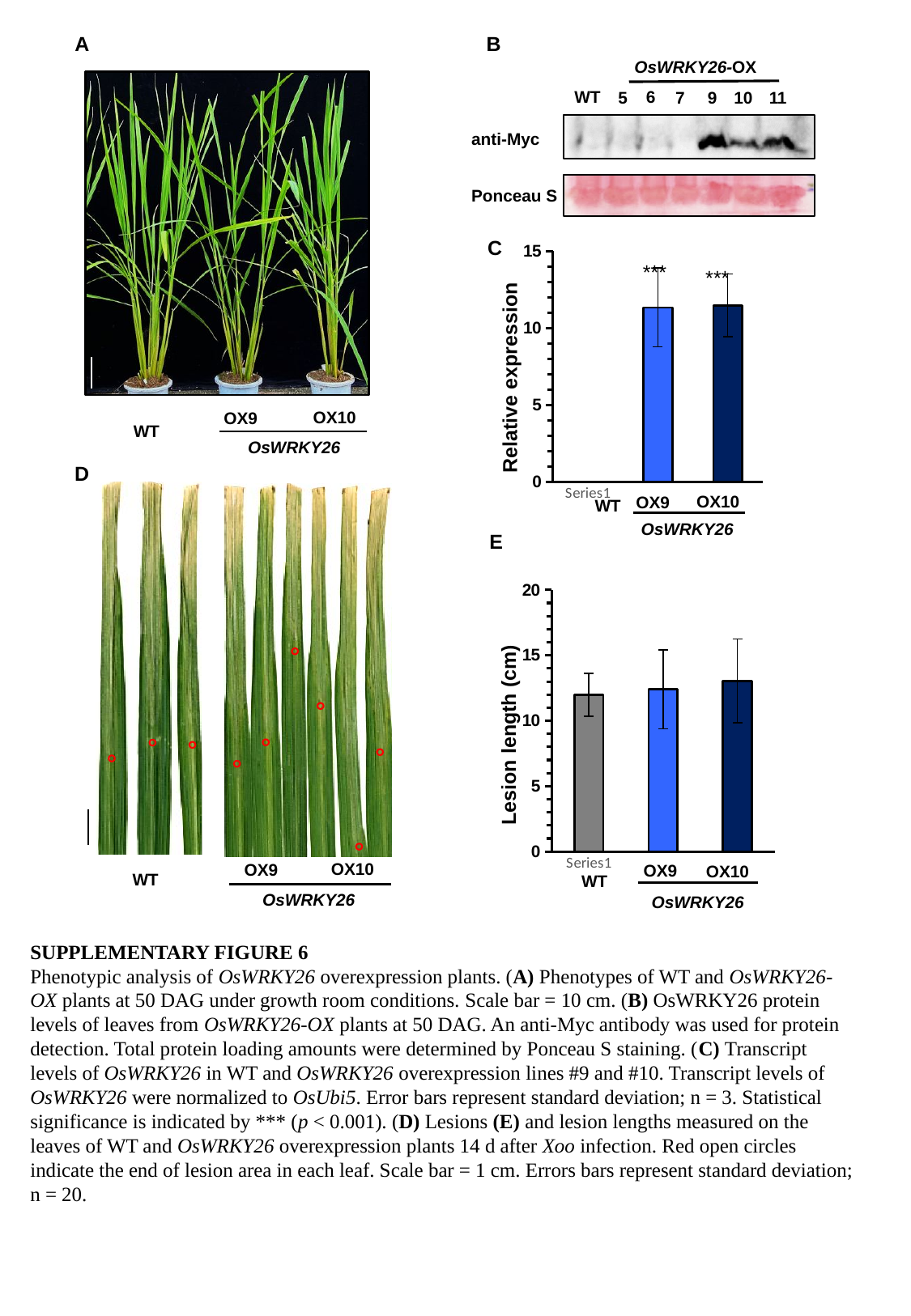

A
B
OsWRKY26-OX
6
WT
5
10
11
7
9
anti-Myc
Ponceau S
C
### Chart
| Category | |
|---|---|
| | 0.023701502277873546 |
| | 11.34254156900981 |
| | 11.4779745682104 |***
***
Relative expression
OX10
OX9
WT
OsWRKY26
D
OX10
OX9
WT
OsWRKY26
OX10
OX9
WT
OsWRKY26
E
### Chart
| Category | |
|---|---|
| | 11.975 |
| | 12.409090909090908 |
| | 13.05 |Lesion length (cm)
OX9
OX10
WT
OsWRKY26
SUPPLEMENTARY FIGURE 6
Phenotypic analysis of OsWRKY26 overexpression plants. (A) Phenotypes of WT and OsWRKY26-OX plants at 50 DAG under growth room conditions. Scale bar = 10 cm. (B) OsWRKY26 protein levels of leaves from OsWRKY26-OX plants at 50 DAG. An anti-Myc antibody was used for protein detection. Total protein loading amounts were determined by Ponceau S staining. (C) Transcript levels of OsWRKY26 in WT and OsWRKY26 overexpression lines #9 and #10. Transcript levels of OsWRKY26 were normalized to OsUbi5. Error bars represent standard deviation; n = 3. Statistical significance is indicated by *** (p < 0.001). (D) Lesions (E) and lesion lengths measured on the leaves of WT and OsWRKY26 overexpression plants 14 d after Xoo infection. Red open circles indicate the end of lesion area in each leaf. Scale bar = 1 cm. Errors bars represent standard deviation; n = 20.

## Slide 8
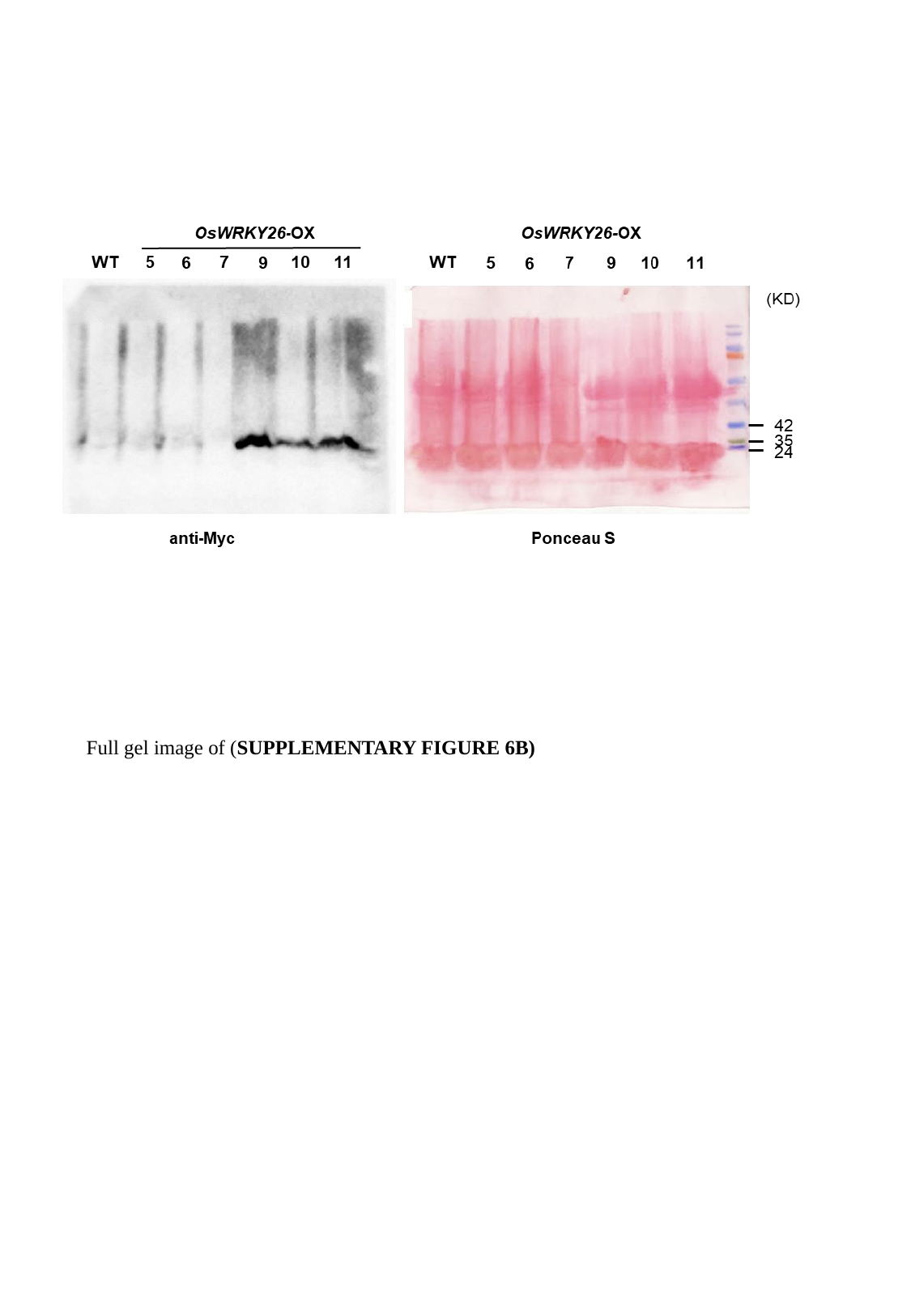

# Full gel image of (SUPPLEMENTARY FIGURE 6B)
